# Electroconvulsive therapy in adult patients with treatment-resistant depression: a systematic review

**DOI:** 10.1590/1806-9282.20251129

**Published:** 2026-05-08

**Authors:** Rui Mateus Joaquim, Paloma Emanoela Fava Felix, Gustavo Henrique Belotto, Ana Luíza da Silva Teles, Leandro Fernandes Malloy-Diniz, Mercêdes Alves, Alfredo Minervino, Rafael Bernardon, Leonardo Baldaçara, Antônio Geraldo da Silva

**Affiliations:** 1 Universidade Federal de Minas Gerais, Saúde Mental Baseada em Evidências – Belo Horizonte (MG), Brazil.; 2 Fundação Dracenense de Educação e Cultura – Dracena (SP), Brazil.; 3 Associação Brasileira de Impulsividade e Patologia Dual – Brasilia (DF), Brazil.; 4 Universidade Federal de Minas Gerais, Faculty of Medicine, Department of Psychiatry – (MG), Brazil.; 5 Associação Brasileira de Psiquiatria – Rio de Janeiro (RJ), Brazil.; 6 Universidade Federal da Paraíba – João Pessoa (PB), Brazil.

## INTRODUCTION

Depression is a chronic and widespread psychiatric disorder, ranking as one of the foremost causes of disability globally. As many as 20% of persons experience a depressive episode in their lifetime, resulting in considerable effects on functioning, quality of life, and suicide risk^[Bibr B1],2,3^. Despite numerous treatment modalities—including antidepressants, psychotherapy, transcranial magnetic stimulation (TMS), and vagus nerve stimulation (VNS)—15–30% of patients fail to attain or maintain sufficient clinical improvement^[Bibr B4],5,6^.

Treatment-resistant depression (TRD) is characterized by an inadequate response to at least two antidepressants from different pharmacological classes^[Bibr B4],7^. It is a significant clinical challenge, characterized by chronicity, disability, and considerable socioeconomic impact. Due to the poor efficacy of traditional treatments, other ­strategies such as pharmacological combinations, esketamine, and brain stimulation therapies have been investigated to enhance outcomes^[Bibr B4],7^.

Electroconvulsive therapy (ECT) is distinguished by its prompt and effective therapeutic response, particularly in severe cases involving suicidal risk or psychotic symptoms^[Bibr B8]^. Nonetheless, its use is limited by high costs, limited availability within the Brazilian public healthcare system, cognitive side effects, and persistent societal stigma. The anesthetic employed can affect both clinical and cognitive outcomes. Consequently, comprehensive investigations are required to assess its efficacy, safety, and customization^[Bibr B7],[Bibr B9]^.

This study aimed to systematically examine randomized controlled clinical trials on ECT for TRD, emphasizing response and remission rates while addressing key limitations and exploring novel therapeutic options.

## METHODOLOGY

### Eligibility criteria

The process of searching for and selecting studies was based on the following research question: “Is there a beneficial contribution of ECT for TRD symptoms for adults aged 18 and over?” It is important to note that this study considered possible interactions between the medications in use and ECT effects.

The inclusion criteria were (i) randomized controlled trials assessing the efficacy of ECT for TRD; (ii) studies that assessed and pre- and post-ECT depression screening measures; and (iii) patients aged 18 years and older.

The exclusion criteria include (i) duplicate studies; (ii) theoretical works; (iii) randomized studies outside the age range; and (iv) insufficient data presentation for the interpretation of the results.

**Figure 1:**
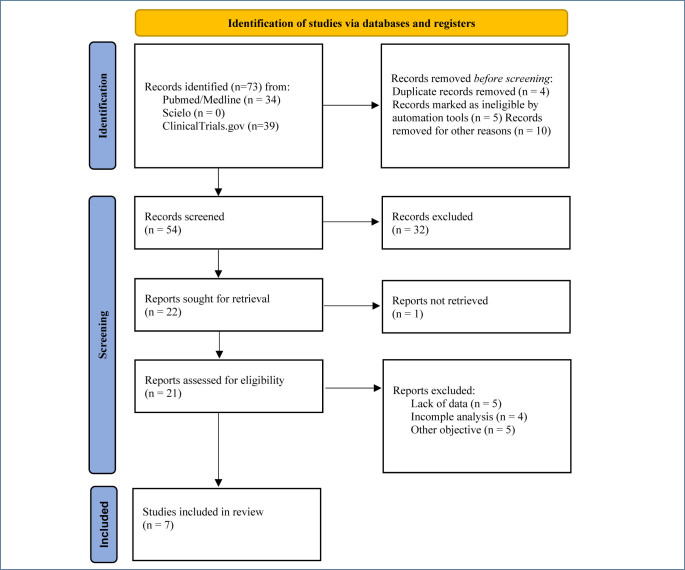
Flowchart. Identification and selection process.

**Figure 2:**
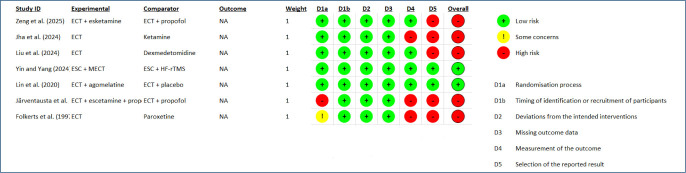
Risk-of-bias assessment.

- **Information sources:** The process of searching and selecting studies was carried out in the PubMed database (Medline), Scielo, and www.clinicaltrials.gov.
- **Search strategy:** The descriptors used for the title and abstract were “treatment-resistant depression” AND “electroconvulsive therapy”. Without the date filter, however, including only randomized studies. For more details, see Figure 1.
- **Selection process:** The study selection process was carried out independently by the authors. First, the titles and abstracts were read, duplicates were excluded, and then the selected studies were read in full to define the final sample. For more details, see Figure 1.
- **Data collection process:** For proper organization of the analysis, data from the studies were transported to Excel sheets, including only patients diagnosed with TRD filling the inclusion criteria, considering gender and education. Intervention strategies were also identified, such as the number of sessions and concomitant medications. In addition, pre- and post-intervention assessments were collected and analyzed, comparing scores for depression between the groups (intervention and control) on scales used to assess depressive symptom intensity before and after intervention. For more details, see Figure 1.
- **Outcomes:** Response and remission. Response was defined as a reduction of at least 50% from baseline scores in the instrument used. Remission was defined as achieving scores below respective cutoffs at the endpoint.
- **Study risk-of-bias assessment:** Robis 2 tool. For more details, see Figure 2.
- **Synthesis:** Data were registered and presented in this manuscript.

### Search and selection of studies

Results of the search and selection process are presented in Figure 1. The process of selection and inclusion of studies took place from March 8 to August 1, 2024. Initially, all titles and abstracts were retrieved and read from chosen databases, duplicates were subsequently excluded, and then a full reading was performed for a final decision. Thus, seven studies were included in the final sample.

All included studies were randomized controlled trials on TRD, and subjects were assessed using validated scales to assess depressive symptoms. Concomitant use of psychotropic medications is a possible confounding factor and was controlled statistically.

### Assessment of the quality of included studies

Studies were independently assessed using the revised Cochrane risk-of-bias tool for randomized trials (RoB 2.0), which includes five main dimensions: (D1) randomization bias, (D2) bias due to deviations from intended interventions, (D3) bias due to missing outcome data, (D4) bias due to outcome measures, and (D5) bias due to selection of reported outcomes.

### Characteristics of included studies

All studies in this systematic review were RCTs and included participants aged 18 and over. Main instruments used to measure outcomes were Hamilton Depression Rating Scale (HDRS), Montgomery-Asberg Depression Rating Scale (MADRS), North American Adult Reading Test-35 (NAART-35, a widely used rapid-administration index for estimating verbal intellectual ability), and Quick Inventory of Depressive Symptomatology Self-Report (QIDS-SR16). Trials tested ECT versus anesthetics, antidepressants, ketamine, or repetitive TMS (rTMS). The number of sessions ranged from 8 to 24.

### Response

In total, seven RCTs were assessed. Response rates varied from 41.2 to 92.7%. Folkerts et al.^10^ reported a 71.4% response rate for ECT and a 27.8% response rate for paroxetine at an interval of 4 weeks. Järventausta et al.^11^ observed comparable reductions in MADRS when employing ECT with esketamine plus propofol or esketamine alone.

In a study by Jha et al. ketamine was shown to be noninferior to ECT in the treatment of depression^12^. Ketamine exhibited greater reductions in QIDS-SR16 by week 2, but the results were comparable at week 3. Ketamine was more effective for outpatients than for inpatients, while ECT was more effective for inpatients. ECT demonstrated a superior response in individuals with very severe depression, high premorbid intelligence, posttraumatic stress disorder (PTSD), or memory impairment.

In both the ECT+agomelatine and ECT+placebo groups, Lin et al.^13^ observed substantial reductions in HAMD-17 (92.7 vs. 90.2%, no significant difference). Liu et al.^14^ found that the posttreatment and follow-up response rates were comparable between the ECT and dexmedetomidine groups.

In comparison to escitalopram (ESC) alone, Yin and Yang^15^ observed that ESC+modified electroconvulsive therapy (MECT) and ESC+high-frequency rTMS (HF-rTMS) resulted in more significant symptoms reduction. However, there was no significant difference in response rates (17, 13, and 12 patients, respectively). According to Zeng et al.^7^, esketamine was not inferior to propofol as an ECT anesthetic, with slightly higher response rates (65 vs. 60%).

### Remission

The review included four RCTs. Remission rates varied from 9.2 to 73.2%. Jha et al.^12^ found that ketamine had a higher rate of remission than ECT among patients with NAART-35<85 (approximately 29% vs. approximately 10%). The differences were less pronounced in individuals with NAART-35 levels that exceeded 85.

According to Lin et al., remission rates with ECT plus agomelatine were comparable to those with ECT plus placebo (73.2 vs. 65.9%, p=0.47)^13^. Liu et al.^14^ reported remission rates of 52.6% in the ECT group and 50% in the dexmedetomidine (DEX) group after 10 sessions. By the end of the study, remission rates were 47.4 and 44.8%, respectively.

In the study of Yin and Yang^15^, ESC+MECT and ESC+HF-rTMS groups showed greater symptom reduction than ESC alone, but no significant difference in remission (17, 13, and 12 patients, respectively; p=0.391).

Zeng et al.^7^ reported that esketamine as an anesthetic had a marginally higher remission rate than propofol (65 vs. 55%) in one comparison and a lower rate in another (60 vs. 65%). Esketamine was not inferior.

## DISCUSSION

In a previous study conducted across three different centers in Brazil, and irrespective of psychiatric diagnosis, the immediate response rate to ECT was 95.8%, while the response rates at 30 and 60 days were 90.6 and 87.7%, respectively^[Bibr B16]^. In this review, we found response rates between 41.2 and 92.7%. Compared to a review by Tokutsu et al., response and remission in TRD rates were 85.7 and 54.8%, respectively. Husain et al. found identical response rates for TRD and non-resistant cases (in both groups 60%)^[Bibr B17]^.

ECT is an expensive treatment despite potentially higher efficacy for the treatment of depression. However, naturalistic studies show a high rate of relapse after discontinuation of ECT^[Bibr B18]^. Few studies assessed long-term follow-up after remission, as Tokutsu et al.^[Bibr B19]^ did. This study followed 34 TRD patients for 1 year after ECT treatment and observed that 52.8% relapsed. The major finding of this study was that elderly patients were more prone to relapse and recurrence, suggesting that older depressed patients may exhibit a greater degree of treatment resistance. One study indicated that, across all diagnoses, a higher number of ECT sessions may be a predictor of better prognosis^[Bibr B16]^. Global predictors of outcome in TRD included comorbid axis I disorders and medical illnesses such as diabetes mellitus and hypertension^[Bibr B19]^.

### Strengths and limitations

The number of clinical trials remains limited, and the sample sizes within each study are generally small. This hinders more in-depth analyses, such as the identification of predictive variables of treatment response. Studies evaluating relapse rates are scarce. The instruments used to assess response and remission are heterogeneous. Finally, various other treatments were associated with ECT, ranging from different antidepressants to various anesthetics. It has not been reported yet whether these patients were undergoing psychotherapy or not. Finally, we report that in the selected RCTs, both the diagnostic criteria for TRD and the outcome measures were well-defined, enabling straightforward extraction of data on treatment response and remission.

## CONCLUSION

There must be greater recognition and investigation of ECT as a treatment option for TRD. ECT should be more widely recognized and studied for TRD. Evidence supports its efficacy, but more diverse, long-term studies comparing ECT to ketamine/esketamine or accelerated rTMS are needed. A clinical framework must guide treatment choices, aiming to reduce relapse and tailor interventions to patient subtypes.

## Data Availability

The datasets generated and/or analyzed during the current study are available from the corresponding author upon reasonable request.
